# Viral and immunologic evaluation of smokers with severe COVID-19

**DOI:** 10.1038/s41598-023-45195-z

**Published:** 2023-10-19

**Authors:** Joseph Vecchio, James Regan, Yuting Jiang, Roy Li, Hannah Romain, Fizah Yousuf, Thomas Adel, Kevin Hall, Jeffrey M. DaCosta, Xu Yu, Jonathan Z. Li, Ismael Ben Fofana

**Affiliations:** 1 Biology Department, Boston College, 140 Commonwealth Avenue, Chestnut Hill, MA USA; 2 Brigham and Women’s Hospital, Harvard Medical School, Boston, MA USA; 3 Ragon Institute of MGH, MIT and Harvard, Harvard Medical School, Cambridge, MA USA

**Keywords:** Immunology, Microbiology

## Abstract

Smoking negatively affects B cell function and immunoglobulin levels, but it is unclear if this immune dysfunction contributes to the risk of severe COVID-19 in smokers. We evaluated binding IgM, IgA and IgG antibodies to spike and receptor binding domain antigens, and used a pseudovirus assay to quantify neutralization titers in a set of 27 patients with severe COVID-19. We found no significant differences between binding and neutralization antibody responses for people with a smoking history and people who never smoked. High plasma viral load, but not antibody titers, was linked to an increased risk of death. Humoral immune dysfunction was not a major driver of severe COVID-19 in smokers.

## Introduction

Severe Acute Respiratory Syndrome 2 (SARS-CoV-2), the etiological agent of coronavirus disease 2019 (COVID-19) emerged in late 2019 in Wuhan (China)^1^. COVID-19 illness is manifested by a wide range of symptoms varying from mild to critical illness^2^. Older patients and those with comorbidities such as diabetes, high blood pressure, coronary heart diseases and chronic kidney diseases present a higher risk of severe disease^3–5^. Cigarette smoking is the major cause of lung cancer and is also associated with diabetes, high blood pressure, cardiovascular diseases (CVD), respiratory diseases, and chronic obstructive pulmonary disease (COPD)^6–9^. Smoking increases angiotensin converting enzyme 2 (ACE2) gene expression in the lungs^10–13^, suggesting a higher potential for SARS-CoV-2 entry into lung cells and an increased risk of severe COVID-19, but studies have produced mixed results^14–17^. Smoking also affects the normal function of B cells^7,10,18^, and smokers were reported to have lower levels of salivary IgA, IgG, and IgM than non-smokers^19^. Tarbiah et al. also found that cigarette smoking differentially affects immunoglobulin class levels in serum and saliva^20^. However, it is unclear to what extent immune dysfunction caused by smoking contributes to the risk of severe COVID-19. In this study, we investigated the associations between smoking history, SARS-CoV-2-specific antibody levels, systemic SARS-CoV-2 RNA levels, cardiovascular and chronic lung disease (CLD), and risk of death. To the best of our knowledge, this is the first study analyzing the joint impact of smoking on antibody responses and plasma viremia levels in COVID-19 patients.

## Methods

### Study enrollment and sample collection

Plasma samples were obtained from the Mass General Brigham Biobank through the Massachusetts Consortium for Pathogen Readiness (MassCPR). Plasma samples were obtained at the time of hospital admission from patients (*n* = 27) with confirmed SARS-CoV-2 infection using RT-PCR and collected between March 2020 and February 2021 at the time of hospital admission. All patients had severe COVID-19 symptoms, but due to variability in disease progression the days post symptom onset of sample collection varied among patients. Initial analyses (see Results) showed that antibody levels generally increased over the first few weeks following symptom onset and then decreased. To explore the impact of this temporal pattern on other analyses, all comparisons were repeated with a reduced data set that removed samples (*n* = 5) collected ≥ 30 days from symptom onset. For the majority of patients only one plasma sample was collected, so here we present the results of single timepoint analyses. Demographics (sex, age and ethnicity), comorbidity (chronic lung disease and cardiovascular disease) information, and smoking status were obtained from patients’ medical records. Pre-COVID-era plasma from 10 naïve individuals were obtained from BIOIVT (Westbury, NY) and included as negative controls for the ELISA and neutralization assays.

### Ethics declaration

This study was approved by the Institutional Review Boards of Boston College (IRB Protocol Number # 21.115.01e) and Mass General Brigham (IRB# 2020P000804). Informed written consent was obtained from all hospitalized participants diagnosed with COVID-19. The authors confirm that all research was performed in accordance with relevant guidelines/regulations.

### Cell lines

Two cell lines were used in this study. Human Kidney Embryonic cells (HEK293T) and HEK293T cells engineered to express the Angiotensin Convertase Enzyme 2 (293T-ACE2)^21^. 293T-ACE2 cells were gifted by Dr. Huihui Mou and Dr. Michael Farzan (SCRIPPS Research Institute, Florida, USA). Both cell lines were cultured in Dulbecco’s modified Eagle’s medium (DMEM) supplemented with fetal bovine serum (FBS) at 10% of the total volume (DMEM10) (ThermoFisher) and containing penicillin/streptomycin (ThermoFisher). Cells were cultured at 5% CO_2_, 37 °C and 70% humidity. ACE2 expression on 293 T-ACE2 was maintained by DMEM10 media supplementation with 3 µg/ml of puromycin dihydrochloride (ThermoFisher). ACE2 expression was confirmed by flow cytometry using anti myc-tag antibody (Abcam)^21^. The myc-tag had previously been fused to human ACE-2 receptor gene during construction of the expression plasmid^21^. Data were acquired using a BD FACSAria flow cytometer (BD Biosciences) and analyzed using FlowJo software (TreeStar) (Supplementary Fig. S1).

### Plasma viral load

SARS-CoV-2 viral load quantification from plasma samples were carried out as previously described^22^. In summary, virions were pelleted from plasma by centrifugation at approximately 21,000 × g for 2 h at 4 °C. The supernatant was removed and TRIzol-LS™ Reagent (ThermoFisher) was added to the pellets and then incubated on ice. Following incubation, chloroform (MilliporeSigma) was added and the sample was vortexed. The mixtures were separated by centrifugation at 21,000 × g for 15 min at 4 °C, and subsequently the aqueous layer was removed and treated with an equal volume of isopropanol (MilliporeSigma). GlycoBlue™ Coprecipitant (ThermoFisher) and 3 M Sodium Acetate (ThermoFisher) were added to each sample and incubated on dry ice until frozen. The RNA was pelleted by centrifugation at 21,000 × g for 45 min at 4 °C. The supernatant was discarded, and the RNA was washed with cold 70% ethanol. The RNA was resuspended in DEPC-treated water (ThermoFisher). Each qPCR reaction contained extracted RNA, 1X TaqPath™ 1-Step RT-qPCR Master Mix, CG (ThermoFisher), the Center for Disease Control CDC N1 forward and reverse primers, and probe (Center for Disease Control). Viral copy numbers were quantified using N1 qPCR standards in 16-fold dilutions to generate a standard curve. The assay was run in triplicate for each sample and two non-template control (NTC) wells were included as negative controls. An internal virion control (RCAS) was spiked into each sample and quantified to determine the efficiency of RNA extraction and qPCR amplification^23^ and each qPCR plate included both SeraCare SARS-CoV-2 pseudoviral positive controls and negative controls.

### SARS-CoV-2 ELISA

ELISA was performed as previously described^24,25^ with slight modifications. Briefly, 96-well Nunc MaxiSorp ELISA plates (Thermo Scientific) were coated with viral antigens diluted in carbonate-bicarbonate buffer to a concentration of 1 µg/mL for all antibody isotypes (IgG, IgA, IgM) before incubation for 1 h at room temperature. Soluble SARS-CoV-2 trimer purchased from Novus Biologicals (Cat # 10,549-CV-100) and soluble receptor binding domain obtained from Innovative Research (Cat # ICOV2RBDRHIS50UG) were used as antigens. After antigen incubation, plates were washed with a buffer consisting of 50 mM Tris (pH 8.0) (ThermoFisher), 140 mM NaCl (MilliporeSigma), and 0.05% Tween-20 (ThermoFisher). Plates were then incubated with a blocking buffer consisting of 1% BSA (MilliporeSigma), 50 mM Tris (pH 8.0), and 140 mM NaCl for 30 min at room temperature, and then washed. Serum samples were diluted 1:100 with a dilution buffer consisting of 1% BSA, 50 mM Tris (pH 8.0), 140 mM NaCl, and 0.05% Tween-20. Samples and standards were added to corresponding wells and incubated at 37 °C for 30 min followed by washing, 5 times. Human antibody isotypes were detected using HRP-conjugated anti-human-IgG, IgM and IgA as secondary antibodies. All secondary antibodies were obtained from ThermoFisher; IgM (Catalog # A18841), IgG (Catalog # 62–8420) and IgA (Catalog # 31,417). The secondary antibodies were diluted as follows: anti-human IgG-HRP (1:4000), anti-human IgM-HRP (1:10,000), and anti-human IgA-HRP (1:4,000). The diluted samples were added to corresponding plates and incubated for 30 min at room temperature. After the washes, TMB substrate (ThermoFisher) was added to each plate for 10 min and the reaction was terminated with TMB stop solution (Southern Biotech). Data were acquired by spectrophotometry at 450 nm using a Victor X5 microplate reader (Perkin Elmer).

### SARS-CoV-2 pseudovirus production

Pseudovirus production and titration were completed using published protocols^25^. All the plasmids used in pseudovirus production were a gift from Dr. Alejandro Balazs. The plasmids were obtained from Addgene under the reference names pHAGE-CMV-luc2-IRES-ZsG-W (Addgene plasmid # 164,432), pRC-CMV-Rev1b (Addgene plasmid # 164,443), pHDM-Tat1b (Addgene plasmid # 164,442), pHDM-Hgpm2 (Addgene plasmid # 164,441), pTwist-SARS-CoV-2 ∆18 (Addgene plasmid # 164,436). Here, 12–15 million HEK293 T cells were seeded in T175 (ThermoFisher) in presence of 25 ml of DMEM10. On the next day, culture media was replaced with a fresh 25 ml DMEM10 before transfection with GenJet (SignaGen Laboratories) following the manufacturer’s recommendations. Twenty-four hours later, transfection media was replaced with fresh DMEM10 and culture supernatant containing secreted pseudoviruses was harvested 3 days post-transfection and cleared using a 0.45 µm Nalgene syringe filter (ThermoFisher). The pseudovirus preparation was divided into 1 ml aliquots per cryovial and stored at − 80 °C.

### SARS-CoV-2 pseudovirus titration

Titration of pseudovirus preparations followed a previously described protocol^25^. Here, 10^4^ 293 T-ACE2 cells were seeded in 100 µl of DMEM10 into 96-well black/clear bottom plates purchased from ThermoFisher (catalog # 165,305). The infection was monitored by visualization and quantification of GFP^+^ cells using an EVOS fluorescence microscope (ThermoFisher). For titration, 2 × serial dilutions of the pseudovirus preparation were tested onto 10^4^ 293 T-ACE2 cells/well. Hundred microliters of diluted pseudovirus preparations were added to corresponding wells. Control (background) wells received 100 µl of DMEM10. After a 72 h-infection period, cells were harvested using trypsin treatment. Infectivity was quantified by detection of GFP^+^ cells using a BD FACSAria flow cytometer (BD Biosciences; Supplementary Fig. S2). Pseudovirus infectivity was also quantified by luciferase assay using duplicate wells. For luciferase assay readout, we opted for the previously described in-house luciferin buffer^25,26^. Assay plates were read using a Victor X5 microplate reader (Perkin Elmer).

### SARS-CoV-2 pseudovirus neutralization assay

Pseudovirus neutralization assays followed a previously described protocol^25^ with modifications for a 96-well plate format. We utilized the ThermoFisher 96-well black/clear bottom plates and monitored infection by visualization and quantification of GFP^+^ cells using an EVOS fluorescent microscope (ThermoFisher). The GFP reporter system was used to monitor infection and determine optimum incubation periods. All reagents, cells, virus and plasma were added in a single streamline with incubation and assay readout in the same plate.

A pseudovirus dilution corresponding of 300 infectious unit based on the Flow Cytometry data was used as viral input. A luciferase readout of 30,000 luminescence rate units (LRU, minimum 10,000 LRU) was targeted as viral input. An incubation period of 84–96 h was preferred for better reproducibility and consistency in our hands. For pseudovirus neutralization assay, patient plasmas were diluted with DMEM10 starting at tenfold dilution and performing 3-time serial dilutions (from 1/10 to 1/21,870). Fifty microliters of diluted plasmas were mixed with 50 µl of pseudovirus dilutions and incubated for 1 h at 37 °C. Thereafter, 10^4^ 293 T-ACE2 cells prepared in 50 µl of DMEM10 were added to virus-plasma mixes. Wells containing cells-only were prepared as assay background while cells plus virus-only (no plasma) were prepared as positive controls corresponding to 100% assay readout. The plates were incubated at 37 °C, 5% CO_2_ and 70% humidity for 84–96 h. Following transduction, cells were lysed and luciferase assay performed as previously described^25,26^. The luciferin buffer was slightly adjusted to obtain a final concentration of 20 mM Tris–HCl (ThermoFisher), 100 mM EDTA (ThermoFisher), 1 mM MgCl_2_ (ThermoFisher), 26.5 mM MgSO_4_ (ThermoFisher), 17 mM dithiothreitol (Goldbio), 250 mM Adenosine-5'-Triphosphate (Goldbio), 750 mM D-luciferin (Goldbio). Fifty microliters of luciferin buffer were added to the well and incubated for 7–10 min and luminescence was quantified within 30 min of buffer addition and after 2 min of shaking using a Victor X5 microplate reader (Perkin Elmer). Neutralization curves were analyzed using GraphPad prism (Supplementary Fig. S3). Neutralizing antibody responses (NT50) were calculated by taking the inverse of the 50% inhibitory concentration value for each sample. Of note, the inverse serial dilution number was multiplied by two to obtain the final NT50 values because (diluted) plasmas were further diluted with equal volumes of pseudovirus during the plasma-virus incubation step.

### Statistical analysis

R v4.2 was used for data and statistical analyses^27^. Based on initial exploration of the data, relationships between the days from symptom onset and either antibody levels or neutralization was visualized by fitting quadratic regression models. Correlations between neutralization and antibody levels were done using nonparametric Spearman’s rank correlation tests. Pairwise comparisons of distributions based on smoking status (former or current versus never), cardiac disease (yes versus no), chronic lung disease (yes versus no), and death (yes versus no) were made using the nonparametric Mann–Whitney *U*-test. To further visualize the association between patient deaths and numerical variables (i.e., antibody levels, viral load, and neutralization), binomial logistic regressions were run with patient outcome (0 = survived, 1 = died) as the response variable. For each family of tests, the false discovery rate method was used to convert *P*-values to *Q*-values and account for running multiple comparisons. Statistical significance was defined as *Q* < 0.05. The relative risk of death was also estimated for smoking status, cardiac disease, chronic lung disease, and viral load detection (no: log10 scores equal 1, yes: log10 scores > 1). In cases with zero observations for a particular category, one was added to all cells.

## Results

Results of the statistical analyses of the full and reduced data sets are presented in the Supplementary data (Supplementary Table S1).

### Clinical characteristic and smoking status

We evaluated hospitalized COVID-19 patients with a smoking history. A total of 17 individuals with a history of smoking was identified and compared to a group of 10 individuals who never smoked (Table 1). These groups were well matched by days from symptom onset (Mann–Whitney *U*-test, *P* > 0.05), percent with CVD, and percent with CLD. However, patients in the ever smoker group were older (Mann–Whitney *U*-test, *P* = 0.02) and biased toward males. Patient demographics (White 63%, Black 15% and Asian 4%) were comparable between groups and were in line with the general US population data. Amongst those with a history of smoking, most were former smokers (82%) and 18% were current smokers. Unfortunately, we do not have complete data on how long ago the former smokers quit nor the rate of smoking (e.g., packs per day) in former/current smokers.

**Table 1:** Demographics and clinical outcomes for participating patients.

	Never smoker (N = 10)	Ever smoker (N = 17)	Total (N = 27)
Female sex, %	60%	12%	30%
Age, median [Q1, Q3]	61.5 [50.75, 67.75]	70 [63, 79]	66 [60.5, 74.5]
Days from symptom onset, median [Q1, Q3]	10.5 [8,25]	18 [14,27]	15 [9,27]
Cardiac disease, %	40%	41%	41%
Chronic lung disease, %	10%	12%	11%
Ethnicities, %			
Caucasian	60%	41%	63%
Black/African	10%	18%	15%
Asian	10%	0%	4%
Other/Unknown	20%	18%	19%
Comorbidities, %			
CLD	10%	12%	11%
CVD	40%	41%	41%
Death rate, %	0%	41%	26%

### General plasma viral load and SARS-CoV-2-specific antibody responses

Although COVID-19 is a respiratory disease, its impact extends beyond the respiratory system and plasma SARS-CoV-2 viremia has previously been described and linked to disease severity^22^. Across all participants, 26% had detectable plasma SARS-CoV-2 viremia, including 10% of non-smokers and 35% of smokers. Detectable viral loads were generally observed during the first 3 weeks after symptom onset (Supplementary Fig. S4).

**Figure 1:**
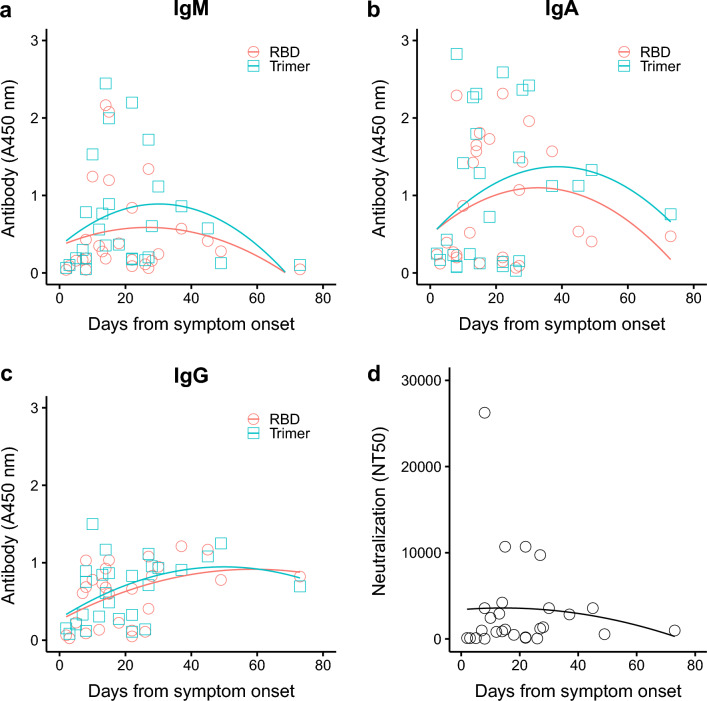
Binding antibodies (IgM, IgA and IgG) and neutralizing antibody levels based on days since symptom onset. Data was obtained using initial specimen collected at admission to the hospital for all 27 patients. (**a**–**c**) IgM, IgA and IgG-specific antibodies detected by ELISA at OD 450 nm. (**d**) Plasma neutralization reported as 50% neutralization titer (NT50). Lines show quadratic regression model fits to each set of data.

COVID-19 disease progression has been associated with spike-specific antibody detection in plasma^24,25,28^. Using ELISA, we detected SARS-CoV-2-specific antibodies (IgM, IgG and IgA) binding to the spike trimer and receptor binding domain (RBD) (Fig. 1a–c). Antibody responses were similar for the trimer and RBD with IgM and IgA displaying a rapid increasing to a peak within the first 3 weeks, before a decrease of IgM levels to near undetectable levels by 8 weeks after symptom onset. Note, however, that there was considerable variation among patient antibody levels for any particular timepoint. IgA levels were lower but more readily detectable for patients who remained hospitalized 6 weeks after symptom onset. IgG responses were relatively slower to develop with higher levels mostly observed between 3 and 6 weeks after symptom onset. Contrary to IgM and IgA responses, IgG were sustained over time and remained comparatively high beyond 6 weeks after symptom onset for people who remained hospitalized.

**Figure 2:**
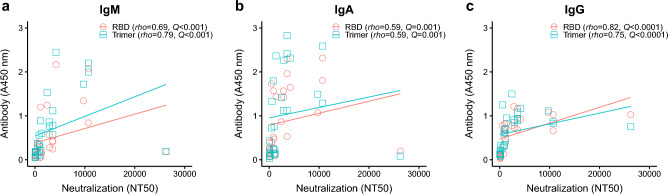
SARS-CoV-2-specific binding antibody levels correlated positively with plasma neutralization titers (NT50). (**a**) NT50 vs IgM, (**b**) NT50 vs IgA, (**c**) NT50 vs IgG. Anti-SARS-CoV-2 RBD and spike trimer antibody levels were compared to their neutralization titers. Correlations were calculated using nonparametric Spearman rank correlation tests. *P*-values were converted to *Q*-values using the false discovery rate to adjust for running multiple tests.

High levels of SARS-CoV2-specific neutralizing antibody responses have previously been correlated with reduced COVID-19 severity^25^. Development of neutralizing antibodies presented a similar trajectory to binding antibodies with higher titers observed within the first 3 weeks (Fig. 1d). One individual presented a super-neutralizing profile with a titer (NT_50_ = 26,244) that was 2.6-fold higher than the next highest titer (NT_50_ = 10,692) (Fig. 1d), albeit with similar binding antibody titers. Interestingly, neutralizing antibody titers were positively correlated with binding antibody titers for all immunoglobulin isotypes and for both spike trimer and RBD antigens (Fig. 2): NT50 vs RBD-IgM (*rho* = 0.69, *Q* < 0.0001), NT50 vs Trimer-IgM (*rho* = 0.79, *Q* < 0.0001), NT50 vs RBD-IgG (*rho* = 0.82, *Q* < 0.0001), and NT50 vs Trimer-IgG (*rho* = 0.75, *Q* < 0.0001). NT50 and IgA-specific responses were also positively correlated, although more modestly than IgM and IgG responses: NT50 vs IgA-RBD (*rho* = 0.59, *Q* = 0.001) or IgA-Trimer (*rho* = 0.59, *Q* = 0.001) (Fig. 2b).

### Smoking status and viro-immunologic parameters

**Figure 3:**
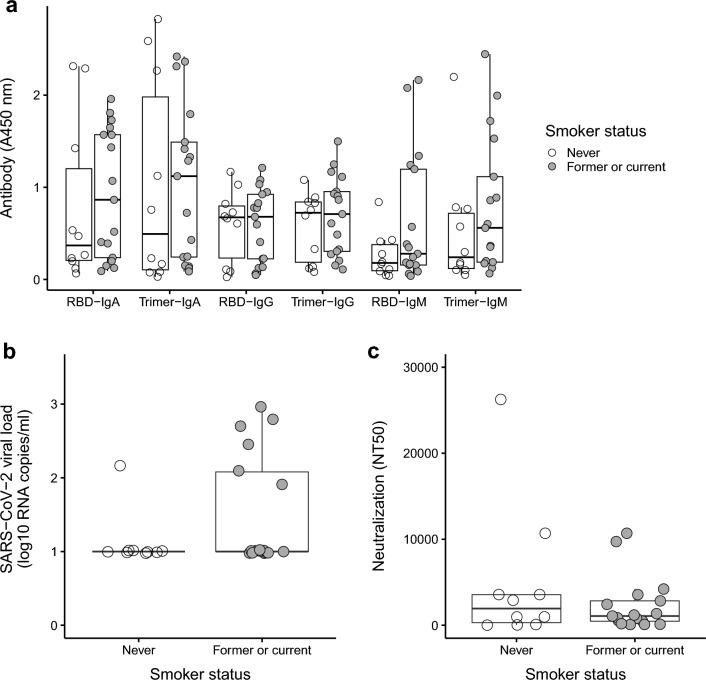
No association between smoking status and viral load or antibodies. Measurements of (**a**) binding antibodies (IgM, IgA and IgG), (**b**) SARS-CoV-2 viral load, and (**c**) neutralizing antibody levels in non-smokers (never smokers) versus smokers (former or current smokers). Comparisons based on smoking status were made using nonparametric Mann–Whitney *U*-tests. All tests were not significant before (*P* > 0.05) and after (*Q* > 0.05) adjusting for multiple tests with the false discovery rate.

We next compared viral and immunologic responses in smokers (former or current, N = 17) vs non-smokers (never smoked, N = 10). There were no statistical differences (*Q*-values > 0.05) between the non-smokers and the smokers in antibody titers (Fig. 3a). However, a trend of higher spike trimer and RBD-binding antibody levels was observed in most comparisons. Although sample size becomes smaller when parsing former (N = 14) and current (N = 3) smokers and comparisons lacked statistical power to detect small effects, average antibody levels tended to be highest in current smokers, intermediate in former smokers, and lowest in never smokers (Supplementary Fig. S5). There was no significant difference (*Q* > 0.05) in both plasma SARS-CoV-2 RNA (Fig. 3b) and neutralizing antibody titers (Fig. 3c) between smokers and non-smokers.

### Viro-immunologic parameters in chronic disease and severe outcomes

There were 11 patients with chronic CVD (seven smokers and four non-smokers), and three patients (two smokers and one non-smoker) with CLD. All three patients with CLD had concurrent CVD. Similar levels (*Q* > 0.05) of binding antibody levels, viremia, and neutralizing antibody titers were detected between individuals with and without CVD (Supplementary Fig. S6). Similarly, no significant differences (Q > 0.05) were found in binding antibodies, viremia, and neutralizing antibody titers in individuals with and without CLD (Supplementary Fig. S7).

**Figure 4:**
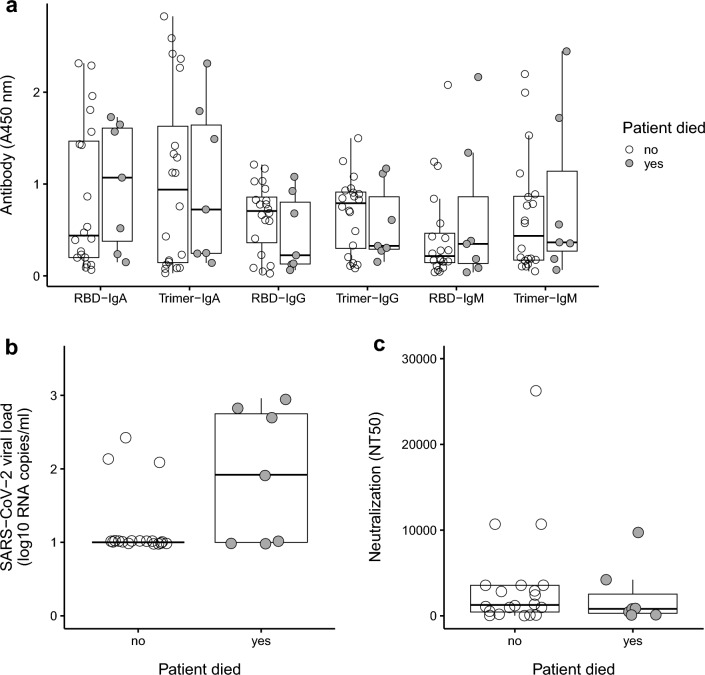
Viremia and antibody responses association with fatality. (**a**) Antibody response (anti-RBD and anti-Trimer IgM, IgA, IgG) in patients who survived and died. (**b**) An initially significant difference (*P* = 0.03) between SARS-CoV-2 viral load in patients who survived versus died did not remain significant (*Q* > 0.05) after adjusting for running multiple tests. (**c**) Neutralization antibodies in patients who survived and died. Comparisons based on patients that survived and died were made using nonparametric Mann–Whitney *U*-tests, with adjustments for multiple tests using the false discovery rate.

**Figure 5:**
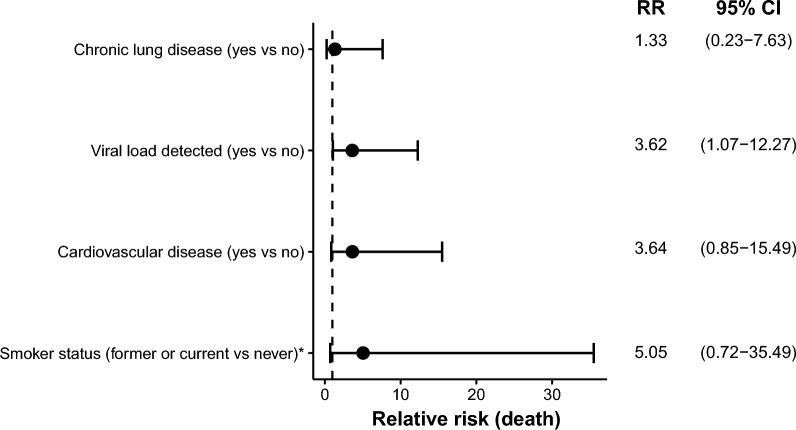
Results mostly consistent with increased relative risk (RR) of death in relation to chronic disease, viral load, and smoking. The dashed line highlights RR = 1, which is the expected outcome with equal risk of death. Analyses with no observed events for one of the comparison groups were marked with an asterisk, and in these cases + 1 was added to each group. All variables had RR estimates > 1 (consistent with increased risk of death) and 95% confidence intervals mostly above 1, but only the RR based on detectable viral load had a confidence interval that was completely above the null expectation.

Seven participants died, all of which had a history of smoking (six former smokers and one current smoker). All participants who died were also male. There were no significant differences in the binding and neutralizing antibody titers between those who died versus those who survived (Fig. 4a,c), but see Supplementary Fig. S8 to view associations via logistic regression. Participants who died had a significantly higher distribution of SARS-CoV-2 RNA viral load compared to the survivors (*U* = 36, *P* = 0.03; Fig. 4b and Supplementary Fig. S8), but this test was no longer significant after adjusting for the false discovery rate (*Q* > 0.05). The relative risk (RR) of death was calculated for CLD, viral load (detected versus not detected), CVD, and smoking status (Fig. 5). All variables had estimated RR > 1, but with wide 95% confidence intervals due to small sample size. While smoking status (former or current versus never) had the highest RR at 5.05, the 95% confidence interval included values below 1 (0.72–35.49). Only viral load detection had a 95% confidence interval that did not include values ≤ 1 [RR 3.62 (1.07–12.27)], which is consistent with a detectable viral load having an increased risk of death.

## Discussion

In this study, we performed viral and humoral immune profiling of a cohort of smokers and non-smokers, while also tracking the potential co-factors of CVD and CLD. No significant correlations were observed between viral load, neutralizing antibody titer and the presence of either CVD or CLD. Despite the relatively small sample size, we found that risk of death was associated with high level SARS-CoV-2 plasma viremia, but not SARS-CoV-2-specific antibody levels.

Smokers are more likely than non-smokers to suffer from lung cancer, diabetes, high blood pressure, cardiovascular diseases (CVD), respiratory diseases, chronic obstructive pulmonary disease (COPD)^6–9^. These conditions also present a higher risk of severe COVID-19 outcome^3–5^. A number of studies have highlighted the association between smoking history and increased risk of COVID-19 disease^14–16,20,29,30^. Vardavas and Nikita reported variabilities between multiple studies but noted that smoking is more likely associated with adverse outcome of COVID-19 progression^29^. Interestingly, Magfira and Helda found a correlation between the prevalence of adult male smoking and the case fatality ratio of COVID-19 in lower middle-income countries (LMIC) but not in upper middle countries and high income countries^30^. Hamer et al.^15^ conducted an analysis of the impact of lifestyle risk factors including smoking on COVID-19 severity using a community-based cohort study of 387,109 adults. They found that obesity as well as lifestyle such as physical inactivity and smoking were associated with a higher risk of severe COVID-19 outcome. Similarly, Del Sole et al.^14^ performed a systematic review of clinical characteristics of severe COVID-19 and found an association with multiple factors including cerebrovascular diseases, chronic obstructive pulmonary diseases, cardiovascular disease and smoking. Another systematic review performed by Reddy et al.^16^ focused specifically on the effect of smoking on COVID-19 severity. The study analyzed 32,849 hospitalized COVID-19 including 25.6% with a smoking history comprising 1501 current smokers, 5676 former smokers and 1240 unspecified smokers. They found that current smokers had an increased risk of severe COVID-19. Patients with a smoking history presented a significantly increased risk of disease progression, severe or critical disease, need for mechanical ventilation and in-hospital mortality. Intriguingly, in our study, all 7 participants who died where subjects with a smoking history, which indicates the need for further investigations with a larger sample size. For instance, the small sample size did not allow for subgroup analysis (e.g., age, gender, ethnicity) for individuals with a smoking history. Of note, all patients who died were Ever smokers but they were also males. The sex bias in severe COVID-19 outcome including higher mortality rates among infected males has been previously reported^31–33^. Unfortunately, to the best of our knowledge, the majority of these previous studies did not assess smoking status as a potential confounding variable. The consideration of both sex and smoking status is therefore warranted for further studies. Our study did not find any significant difference in plasma antibody levels between smokers and non-smoker which is in contrast with previous finding by Tarbiah et al.^20^. We intend to apply these analyses to a larger sample size including non-hospitalized COVID-19 patients.

In summary, we performed a detailed assessment of SARS-CoV-2 viral load, IgG, IgM and IgA binding antibodies, and neutralizing antibody titers in smokers and non-smokers with COVID-19. We found no significant differences between binding and neutralization antibody responses for people with a smoking history and people who never smoked. High plasma viral load, but not antibody titers, was linked to an increased risk of death. The results suggest that humoral immune dysfunction is not a major driver of COVID-19-related mortality in smokers.

### Supplementary Information

Supplementary Information.

## Data Availability

Data presented in this study are available by request to the corresponding authors.
