# Supplementary material for: Viral and immunologic evaluation of smokers with severe COVID-19
Source: Sci Rep. 2023 Oct 19;13:17898. doi: 10.1038/s41598-023-45195-z (PMC10587108; doi:10.1038/s41598-023-45195-z)
Supplement: Supplementary file 1 — Supplementary Information. [file 41598_2023_45195_MOESM1_ESM.docx]

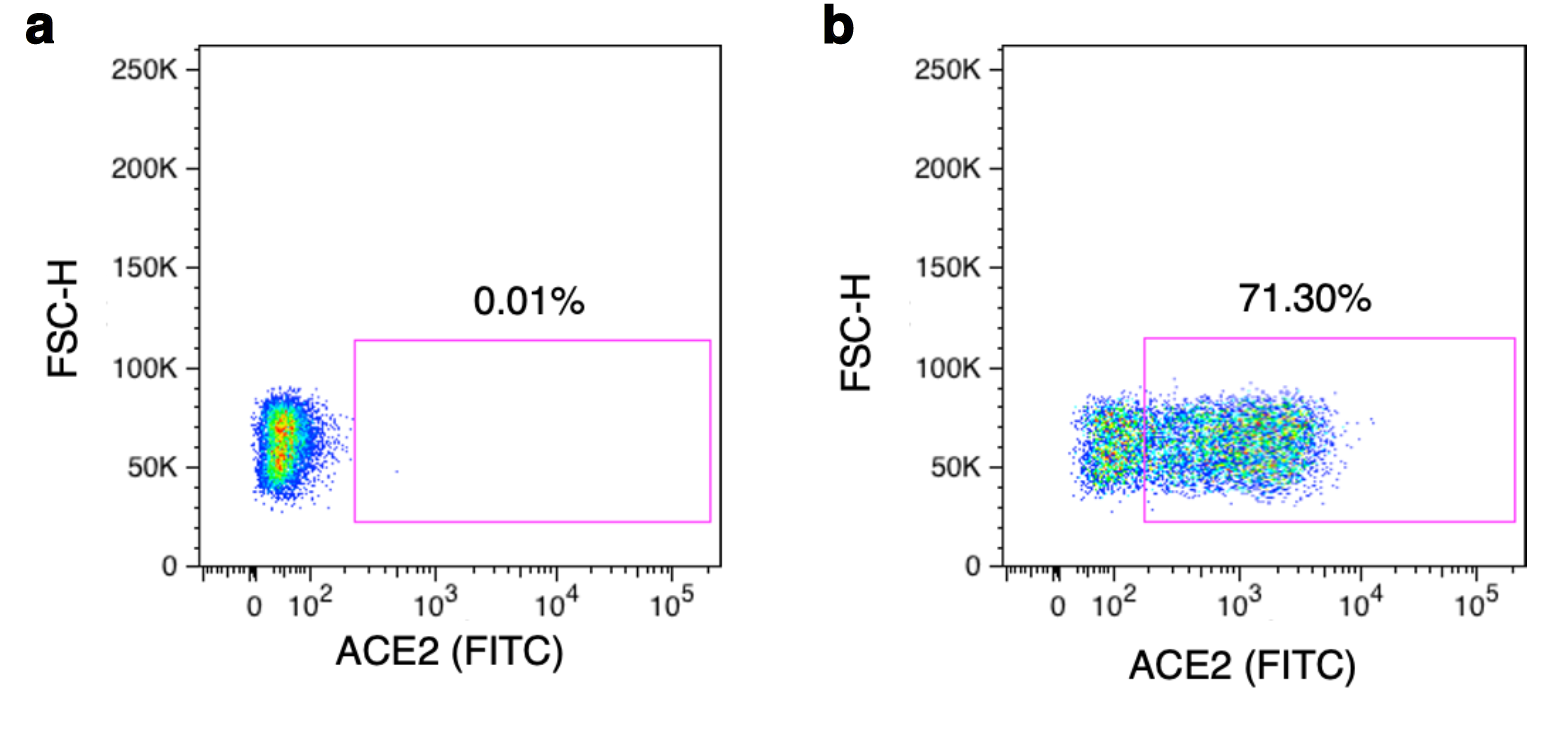


**Supplementary Figure S1.** **Surface expression of Angiotensin-converting enzyme 2 (ACE2).** Target cells were stained with FITC-conjugated anti-myc-tag and ACE2 (FITC) positive cells detected by flow cytometry. Data was analyzed using FlowJo software. The left panel correspond to a control sample with 293T-cells. The right panel correspond to 293TACE2 target cells. FITC: Fluorescein isothiocyanate. FSC-H: Forward Scatter Height.


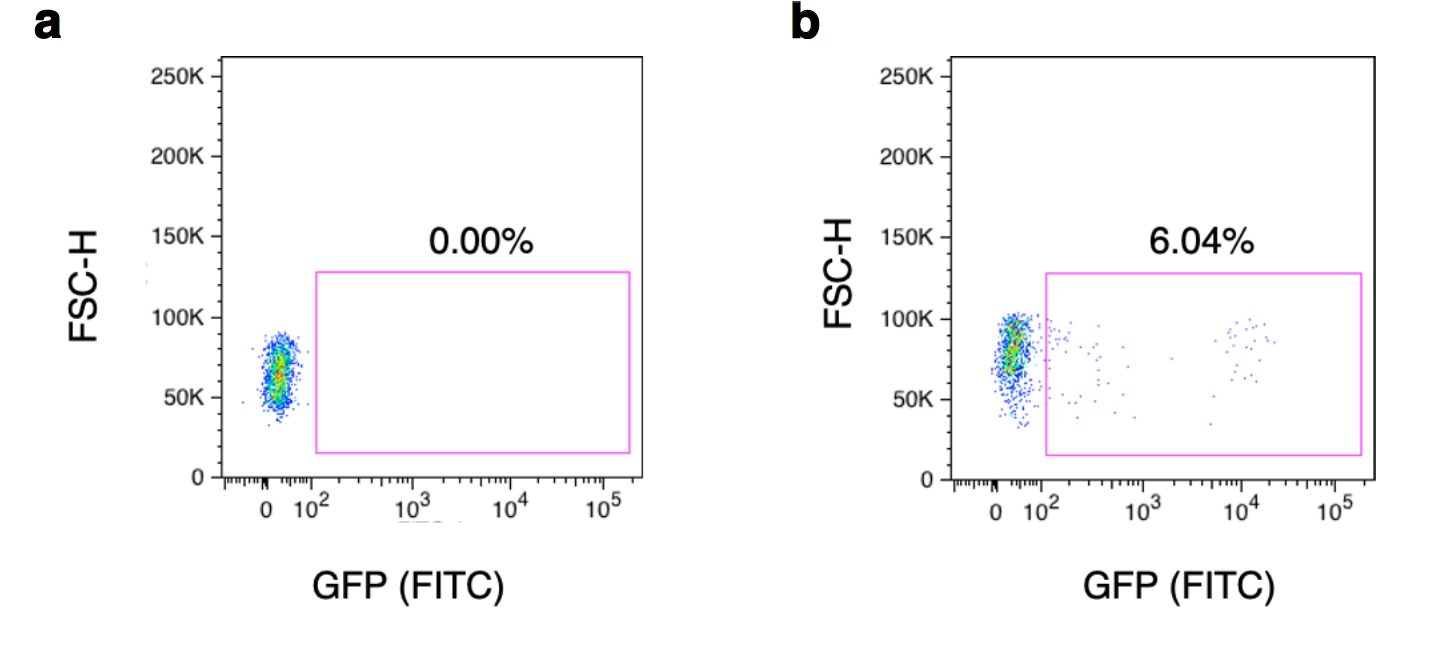


**Supplementary Figure S2.** **Pseudovirus titration using 293-ACE2 target cells.** Cells were infected with serial dilutions of the pseudovirus preparation. The number of infectious units (IU) correspond to the percentage of GFP positive cells. The left panel represents a control sample with no virus addition. The right panel correspond a 2x dilution of this pseudovirus preparation. GFP: Green Fluorescent Protein. FITC: Fluorescein isothiocyanate. FSC-H: Forward Scatter Height.


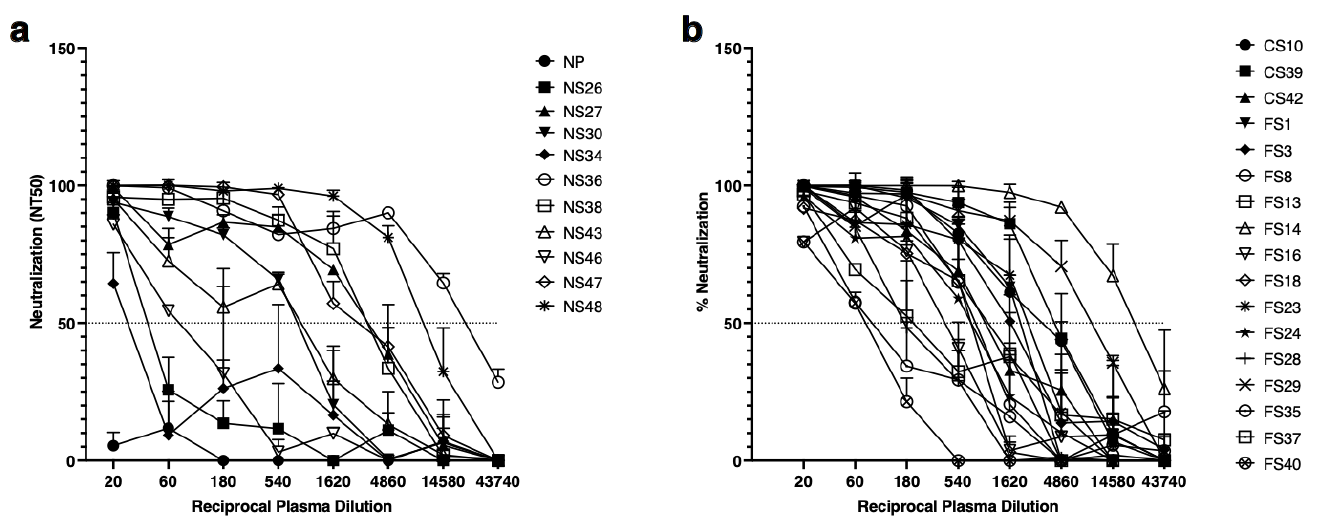


**Supplementary Figure S3**. **Plasma neutralization antibody assay.** Serial dilutions of patient plasmas were tested for neutralization of SARS-CoV-2 pseudovirus preparation. The plasma dilutions were incubated with equal amount of pseudovirus and tested for a single round infection of 293T-ACE2 target cells. Data are representative of two independent experiments. Patients who never smoked are presented in panel “a” and smokers (former and current) are presented in panel “b”. Dotted lines represent 50% neutralization. A pool of 10 naïve plasma “NP” from pre-pandemic non-infected individual was used as a negative control.


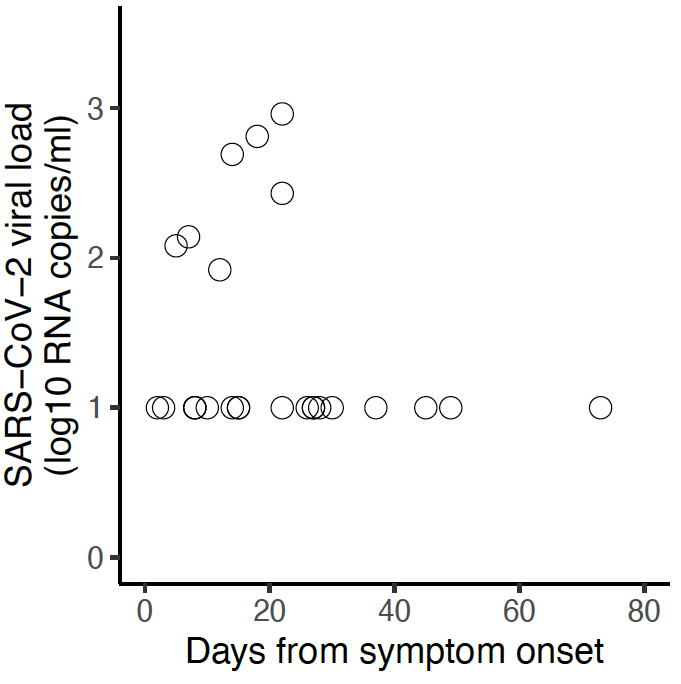


**Supplementary Figure S4. SARS-CoV-2 viral load levels based on days from symptom onset.** Viral load levels were quantified by qPCR, and values above 1 were considered detectable.


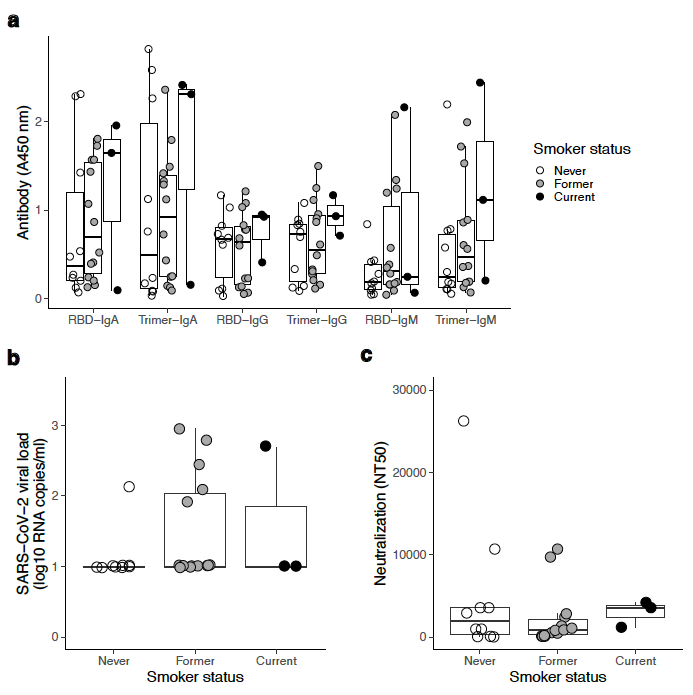


**Supplementary Figure S5.** **Viremia and antibody responses association with smoker status.** In contrast to Figure 3, the status of “ever” smokers is parsed into former and current smoker categories. **(a)** Antibody response (anti-RBD and anti-Trimer IgM, IgA, IgG), **(b)** SARS-CoV-2 viral load, and **(c)** neutralization antibodies based on smoking status.


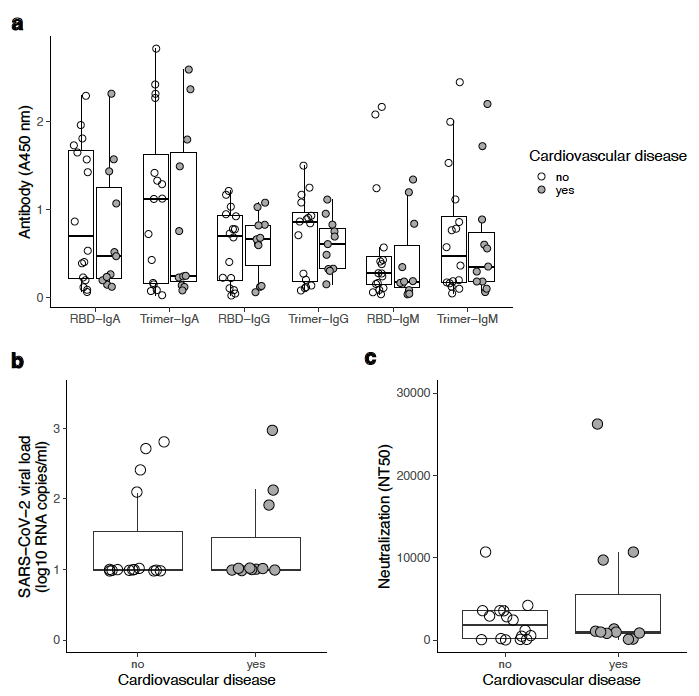


**Supplementary Figure S6.** **Viremia and antibody responses association with cardiovascular disease. (a)** Antibody response (anti-RBD and anti-Trimer IgM, IgA, IgG), **(b)** SARS-CoV-2 viral load, and **(c)** neutralization antibodies based on the presence or absence of cardiovascular disease. All comparisons using nonparametric Mann-Whitney *U*-tests were not significant, before and after adjustments for multiple tests using the false discovery rate.


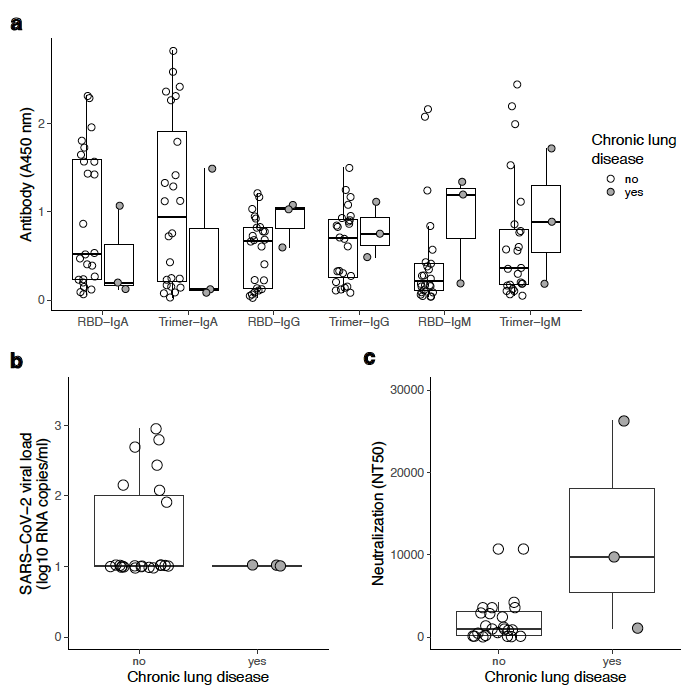


**Supplementary Figure S7.** **Viremia and antibody responses association with chronic lung disease. (a)** Antibody response (anti-RBD and anti-Trimer IgM, IgA, IgG), **(b)** SARS-CoV-2 viral load, and **(c)** neutralization antibodies based on the presence or absence of chronic lung disease. All comparisons using nonparametric Mann-Whitney *U*-tests were not significant, before and after adjustments for multiple tests using the false discovery rate.


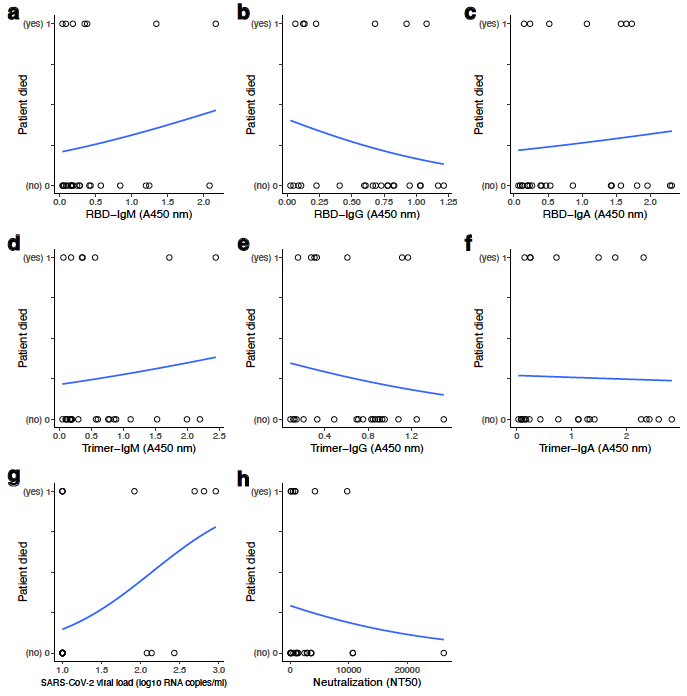


**Supplementary Figure S8. Logistic regression analyses of associations between viremia and antibody responses with patient death.** Patient death was coded as a binary response variable (0 = no, 1 = yes), with explanatory variables of (a-f) anti-RBD and anti-Trimer IgM, IgA, IgG, (g) viral load, and (h) neutralization antibodies. Only the model analyzing viral load was initially significant (*P* = 0.03), but this result became nonsignificant (*Q* > 0.05) after adjusting for multiple tests using the false discovery rate.

**Table S1 Comparison of statistical analysis results based on the full data set (*n*=27) and the reduced data set (*n*=22) with the removal of samples that were collected ≥ 30 days from symptom onset.** Bold terms highlight *P* or *Q* values that are below 0.05.

|  |  |  | **Full data set** | | | **Reduced data set** | | |
| --- | --- | --- | --- | --- | --- | --- | --- | --- |
| **Analysis** | **X** | **Y** | **Statistic*** | ***P*** | ***Q*** | **Statistic*** | ***P*** | ***Q*** |
| Spearman's rank cor. | NT50 | RBD-IgM | 0.695 | **5.8E-05** | **<0.001** | 0.729 | **1.2E-04** | **<0.001** |
| Spearman's rank cor. | NT50 | Trimer-IgM | 0.785 | **1.2E-06** | **<0.001** | 0.794 | **1.0E-05** | **<0.001** |
| Spearman's rank cor. | NT50 | RBD-IgA | 0.592 | **0.001** | **0.003** | 0.550 | **0.008** | **0.008** |
| Spearman's rank cor. | NT50 | Trimer-IgA | 0.593 | **0.001** | **0.003** | 0.558 | **0.007** | **0.008** |
| Spearman's rank cor. | NT50 | RBD-IgG | 0.820 | **1.7E-07** | **<0.001** | 0.882 | **5.8E-08** | **<0.001** |
| Spearman's rank cor. | NT50 | Trimer-IgG | 0.752 | **6.2E-06** | **<0.001** | 0.856 | **3.7E-07** | **<0.001** |
| Mann-Whitney | Died | RBD-IgM | 63 | 0.725 | 0.910 | 46 | 0.680 | 0.957 |
| Mann-Whitney | Died | RBD-IgG | 84 | 0.464 | 0.910 | 54 | 0.945 | 1.000 |
| Mann-Whitney | Died | RBD-IgA | 57 | 0.498 | 0.910 | 39 | 0.368 | 0.910 |
| Mann-Whitney | Died | Trimer-IgM | 65 | 0.808 | 0.910 | 48 | 0.783 | 0.957 |
| Mann-Whitney | Died | Trimer-IgG | 78 | 0.685 | 0.910 | 51 | 0.945 | 1.000 |
| Mann-Whitney | Died | Trimer-IgA | 64 | 0.766 | 0.910 | 41 | 0.448 | 0.910 |
| Mann-Whitney | Died | NT50 | 81 | 0.561 | 0.910 | 58 | 0.724 | 0.957 |
| Mann-Whitney | Died | VL | 36 | **0.026** | 0.436 | 28.5 | 0.075 | 0.718 |
| Mann-Whitney | Died | Age | 25 | **0.014** | 0.436 | 22 | **0.034** | 0.718 |
| Mann-Whitney | Smoker | RBD-IgM | 63 | 0.286 | 0.910 | 45 | 0.482 | 0.910 |
| Mann-Whitney | Smoker | RBD-IgG | 79 | 0.786 | 0.910 | 50 | 0.714 | 0.957 |
| Mann-Whitney | Smoker | RBD-IgA | 72 | 0.537 | 0.910 | 50 | 0.714 | 0.957 |
| Mann-Whitney | Smoker | Trimer-IgM | 59 | 0.204 | 0.910 | 41 | 0.330 | 0.910 |
| Mann-Whitney | Smoker | Trimer-IgG | 66 | 0.360 | 0.910 | 45 | 0.482 | 0.910 |
| Mann-Whitney | Smoker | Trimer-IgA | 73 | 0.570 | 0.910 | 48 | 0.616 | 0.957 |
| Mann-Whitney | Smoker | NT50 | 90 | 0.821 | 0.910 | 57 | 0.973 | 1.000 |
| Mann-Whitney | Smoker | VL | 57 | 0.189 | 0.910 | 34 | 0.198 | 0.910 |
| Mann-Whitney | CVD | RBD-IgM | 102 | 0.512 | 0.910 | 61 | 0.974 | 1.000 |
| Mann-Whitney | CVD | RBD-IgG | 89 | 0.981 | 1.000 | 49 | 0.497 | 0.910 |
| Mann-Whitney | CVD | RBD-IgA | 97 | 0.680 | 0.910 | 60 | 1.000 | 1.000 |
| Mann-Whitney | CVD | Trimer-IgM | 91 | 0.904 | 0.962 | 55 | 0.771 | 0.957 |
| Mann-Whitney | CVD | Trimer-IgG | 97 | 0.680 | 0.910 | 52 | 0.628 | 0.957 |
| Mann-Whitney | CVD | Trimer-IgA | 93 | 0.827 | 0.910 | 57 | 0.872 | 1.000 |
| Mann-Whitney | CVD | NT50 | 78.5 | 0.657 | 0.910 | 46.5 | 0.391 | 0.910 |
| Mann-Whitney | CVD | VL | 83 | 1.000 | 1.000 | 59.5 | 0.737 | 0.957 |
| Mann-Whitney | CLD | RBD-IgM | 17 | 0.162 | 0.910 | 13 | 0.160 | 0.910 |
| Mann-Whitney | CLD | RBD-IgG | 19 | 0.215 | 0.910 | 10 | 0.087 | 0.718 |
| Mann-Whitney | CLD | RBD-IgA | 51 | 0.278 | 0.910 | 39 | 0.356 | 0.910 |
| Mann-Whitney | CLD | Trimer-IgM | 24 | 0.393 | 0.910 | 20 | 0.464 | 0.910 |
| Mann-Whitney | CLD | Trimer-IgG | 28 | 0.583 | 0.910 | 18 | 0.356 | 0.910 |
| Mann-Whitney | CLD | Trimer-IgA | 50 | 0.313 | 0.910 | 39 | 0.356 | 0.910 |
| Mann-Whitney | CLD | NT50 | 13 | 0.082 | 0.905 | 10 | 0.085 | 0.718 |
| Mann-Whitney | CLD | VL | 45 | 0.304 | 0.910 | 37.5 | 0.231 | 0.910 |

*Statistic: Spearman’s rank correlation = *ρ*, Mann-Whitney *U*-test = *U*.
